# Supplementary material for: Lightweight wavelet-CNN tea leaf disease detection
Source: PLoS One. 2025 May 27;20(5):e0323322. doi: 10.1371/journal.pone.0323322 (PMC12112342; doi:10.1371/journal.pone.0323322)
Supplement: Attachment — Submitted filename: Response to Reviewers.docx [file pone.0323322.s003.docx]

**PONE-D-24-43399**

**Lightweight wavelet-CNN tea leaf disease detection**

**Response Letter to Reviewers' Comments**

**Comments from the Editor:**

Thank you for submitting your manuscript to PLOS ONE. After careful consideration, we feel that it has merit but does not fully meet PLOS ONE’s publication criteria as it currently stands. Therefore, we invite you to submit a revised version of the manuscript that addresses the points raised during the review process.

**Response:**

Dear Dr. Rahman,

We sincerely appreciate the opportunity to revise and resubmit our manuscript titled “Lightweight wavelet-CNN tea leaf disease detection” (PONE-D-24-43399R1) to PLOS ONE. We are grateful to the reviewers and the academic editor for their constructive comments and valuable suggestions, which have helped us improve the quality and clarity of our work.

In the revised manuscript, we have carefully addressed all the points raised during the review process. Below, we provide a detailed response to each comment, outlining the modifications made accordingly. We have also included the following required documents:

Response to Reviewers – A point-by-point response detailing the revisions made.

Revised Manuscript with Track Changes – A marked-up version highlighting all modifications.

Clean Revised Manuscript – A final version without tracked changes.

We have ensured that our revisions align with PLOS ONE's publication criteria and hope that the revised manuscript now meets the journal’s standards. We appreciate your time and consideration and look forward to your feedback.

**Response to Journal Requirements**

Journal Requirements:

Please review your reference list to ensure that it is complete and correct. If you have cited papers that have been retracted, please include the rationale for doing so in the manuscript text, or remove these references and replace them with relevant current references. Any changes to the reference list should be mentioned in the rebuttal letter that accompanies your revised manuscript. If you need to cite a retracted article, indicate the article’s retracted status in the References list and also include a citation and full reference for the retraction notice.

**Response:**

We have carefully reviewed all references in our manuscript to ensure their completeness and accuracy. Considering factors such as accessibility, academic relevance, and innovation, we have replaced ten references with more appropriate and up-to-date sources. The specific changes are as follows:

1、Original Reference:

Zhang G, Lyu Z, Liu H, et al. Model for identifying lotus leaf pests and diseases using improved DenseNet and transfer learning. Trans Chinese Soc Agric Eng. 2023;39:188-196.

Replaced with:

Chen J, Dong C, Kong R, Wei Y, Yao H, Zhao Y, et al. Research on identification algorithm of crop pests and diseases based on improved DenseNet model. Eur J Agron. 2023/06/08;12707. doi: 10.1117/12.2681193.

2、Original Reference:

Hossain M S, Mou R M, Hasan M M, et al. Recognition and detection of tea leaf’s diseases using support vector machine. In: 2018 IEEE 14th International Colloquium on Signal Processing & Its Applications (CSPA); 2018:150-154.

Replaced with:

Lin L-H, Li C-H, Yang S, Li S-Z, Wei Y. Automated classification of Wuyi rock tealeaves based on support vector machine. Concurrency and Computation: Practice and Experience. 2019/12/10;31(23). doi: 10.1002/cpe.4519.

3、Original Reference:

Wang R, Chen F, Zhu X, et al. Identifying apple leaf diseases using improved EfficientNet. Transactions of the Chinese Society of Agricultural Engineering (Transactions of the CSAE). 2023;39(18):201-210.

Replaced with:

Xiao H, Shen H, Zhu H, Ye J, Deng S, Xiao Z. Apple Leaf Pathology Detection Based on Improved EfficientNetV2 | IEEE Conference Publication | IEEE Xplore. 2023 IEEE 3rd International Conference on Electronic Communications, Internet of Things and Big Data (ICEIB). 2023. doi: 10.1109/ICEIB57887.2023.10170523.

4、Original Reference:

Li Z, Xu J, Zheng L, et al. Small sample recognition method of tea disease based on improved DenseNet. Transactions of the Chinese Society of Agricultural Engineering (Transactions of the CSAE). 2022;38(10):182-190.

Replaced with:

Deng X, Photong C. Evaluation of Tea Leaf Disease Identification Based on Convolutional Neural Networks VGG16, ResNet50, and DenseNet169 Image Recognitions | IEEE Conference Publication | IEEE Xplore. 2024 12th International Electrical Engineering Congress (iEECON). 2024. doi: 10.1109/iEECON60677.2024.10537865.

5、Original Reference:

Liu Y, Feng Q, Wang S. Plant disease identification method based on lightweight CNN and mobile application. Transactions of the Chinese Society of Agricultural Engineering (Transactions of the CSAE). 2019;35(17):194-204.

Replaced with:

Bhuyan P, Singh PK, Das SK, Bhuyan P, Singh PK, Das SK. Res4net-CBAM: a deep cnn with convolution block attention module for tea leaf disease diagnosis. Multimedia Tools and Applications. 2023-11-03;83(16). doi: 10.1007/s11042-023-17472-6.

6、Original Reference:

Wang Z, Ma F, Zhang Y, et al. Crop disease recognition using attention mechanism and multi-scale lightweight network. Transactions of the Chinese Society of Agricultural Engineering (Transactions of the CSAE). 2022;38(Suppl):176-183.

Replaced with:

Chen J, Chen W, Zeb A, Zhang D, Nanehkaran YA, Chen J, et al. Crop pest recognition using attention-embedded lightweight network under field conditions. Applied Entomology and Zoology. 2021-03-30;56(4). doi: 10.1007/s13355-021-00732-y.

7、Original Reference:

Huang L, Qian B, Guan F, et al. Goat face recognition model based on wavelet transform and convolutional neural networks. Trans Chin Soc Agric Mach. 2023;54(5):278-87.

Replaced with:

Fujieda S, Takayama K, Hachisuka T. Wavelet Convolutional Neural Networks for Texture Classification. arXiv. 2017/07/24. doi: 10.48550/arXiv.1707.07394.

8、Original Reference:

Jia Z, Zhang Y, Wang H, et al. Identification method of tomato disease period based on Res2Net and bilinear attention mechanism. Trans Chinese Soc Agric Mach. 2022;53(7):259-66.

Replaced with:

Zhao Y, Chen J, Xu X, Lei J, Zhou W. SEV‐Net: Residual network embedded with attention mechanism for plant disease severity detection. Concurrency and Computation: Practice and Experience. 2021/05/25;33(10). doi: 10.1002/cpe.6161.

9、Original Reference:

Peng H, Xu H, Liu H, et al. Model for identifying grape pests and diseases based on two-branch feature fusion and attention mechanism. Trans Chinese Soc Agric Eng. 2022;38(10):156-65.

Replaced with:

Xia S, Kou J, Liu N, Yin T, Xia S, Kou J, et al. Scene text recognition based on two-stage attention and multi-branch feature fusion module. Applied Intelligence. 2022-10-22;53(11). doi: 10.1007/s10489-022-04241-5.

10、Original Reference:

Sun J, Zhu W, Luo Y, et al. Recognizing the diseases of crop leaves in fields using improved MobileNet-V2. Transactions of the Chinese Society for Agricultural Machinery (Transactions of the CSAE). 2021;37(22):161-9.

Replaced with:

Ratha A, Barpanda N, Sethy P, Behera S. Automated Classification of Indian Mango Varieties Using Machine Learning and MobileNet-v2 Deep Features. Traitement du Signal. 2024;41(2). doi: 10.18280/ts.410210.

To enhance readability and facilitate access for readers, we have also added the DOI for each reference where applicable. These changes have been incorporated into the revised manuscript, and a summary of the modifications is provided in the rebuttal letter.

Additional Editor Comments:

1. Sections, Sub Sections are not in good format. Such Material and Method (1.,1.1, 1.1.1) etc.

2. Some Figures are not transparent such 3, 5 etc.

3. Follow the overall standard format of the Research article according to the PLOS ONE Criteria.

**Response:**

Dear Editor,

Thank you for your valuable feedback. We have carefully reviewed and revised the manuscript to address the formatting and figure-related concerns:

1、Formatting Issues: We have corrected the section and subsection numbering (e.g., "1., 1.1, 1.1.1") to ensure consistency and adherence to the standard format required by PLOS ONE. Additionally, we have reviewed and adjusted other formatting elements, such as headings, page numbers, and line spacing.

2、Figure Transparency and Clarity: To improve the clarity of Figures 3 and 5, we have reprocessed and enhanced the images to ensure that they are clear and meet the journal’s quality standards.

3、Overall Formatting Compliance: The entire manuscript has been revised to align with the standard research article format outlined by PLOS ONE. We have ensured consistency in text formatting, figure placement, and overall presentation.

We appreciate the time and effort you and the reviewers have invested in evaluating our work. We look forward to your further feedback.
